# Quantitative SWATH-based proteomic profiling of urine for the identification of endometrial cancer biomarkers in symptomatic women

**DOI:** 10.1038/s41416-022-02139-0

**Published:** 2023-02-17

**Authors:** Kelechi Njoku, Andrew Pierce, Bethany Geary, Amy E. Campbell, Janet Kelsall, Rachel Reed, Alexander Armit, Rachel Da Sylva, Liqun Zhang, Heather Agnew, Ivona Baricevic-Jones, Davide Chiasserini, Anthony D. Whetton, Emma J. Crosbie

**Affiliations:** 1 Division of Cancer Sciences, University of Manchester, School of Medical Sciences, Faculty of Biology, Medicine and Health, 5th Floor Research, St Mary’s Hospital, Oxford Road, Manchester, M13 9WL UK; 2 Stoller Biomarker Discovery Centre, Institute of Cancer Sciences, Faculty of Biology, Medicine and Health, University of Manchester, Manchester, UK; 3 Department of Obstetrics and Gynaecology, Manchester University NHS Foundation Trust, Manchester Academic Health Science Centre, Manchester, UK; 4 School of Medical and Health Sciences, College of Human Sciences, Fron Heulog, Bangor University, Bangor, Gwynedd, LL57 2TH UK; 5 Department of Medicine and Surgery, Section of Physiology and Biochemistry, University of Perugia, 06132 Perugia, Italy; 6 School of Veterinary Medicine, Faculty of Health and Medical Sciences, University of Surrey, Guildford, GU2 7XH UK

**Keywords:** Tumour biomarkers, Endometrial cancer

## Abstract

**Background:**

A non-invasive endometrial cancer detection tool that can accurately triage symptomatic women for definitive testing would improve patient care. Urine is an attractive biofluid for cancer detection due to its simplicity and ease of collection. The aim of this study was to identify urine-based proteomic signatures that can discriminate endometrial cancer patients from symptomatic controls.

**Methods:**

This was a prospective case–control study of symptomatic post-menopausal women (50 cancers, 54 controls). Voided self-collected urine samples were processed for mass spectrometry and run using sequential window acquisition of all theoretical mass spectra (SWATH-MS). Machine learning techniques were used to identify important discriminatory proteins, which were subsequently combined in multi-marker panels using logistic regression.

**Results:**

The top discriminatory proteins individually showed moderate accuracy (AUC > 0.70) for endometrial cancer detection. However, algorithms combining the most discriminatory proteins performed well with AUCs > 0.90. The best performing diagnostic model was a 10-marker panel combining SPRR1B, CRNN, CALML3, TXN, FABP5, C1RL, MMP9, ECM1, S100A7 and CFI and predicted endometrial cancer with an AUC of 0.92 (0.96–0.97). Urine-based protein signatures showed good accuracy for the detection of early-stage cancers (AUC 0.92 (0.86–0.9)).

**Conclusion:**

A patient-friendly, urine-based test could offer a non-invasive endometrial cancer detection tool in symptomatic women. Validation in a larger independent cohort is warranted.

## Introduction

Endometrial cancer is the most common gynaecological malignancy in high-income countries and the sixth most common cancer in women worldwide. In 2020, there were over 400,000 incident cases and 97,000 endometrial cancer-related deaths globally [1]. The incidence of endometrial cancer is rising rapidly, a consequence of the growing obesity epidemic and an ageing population [2]. When diagnosed early, endometrial cancer is amenable to curative surgical resection and has a favourable prognosis [3]. A significant minority of women, however, present with high risk or advanced disease and have poor outcomes [4, 5]. Novel approaches that facilitate the early detection of endometrial cancer have potential to improve the outlook of women with biologically aggressive disease [3].

Post-menopausal bleeding (PMB) is a red flag symptom of endometrial cancer that triggers urgent investigation by sequential invasive tests, including transvaginal ultrasound scan (TVS), hysteroscopy and endometrial biopsy. These tests aim to identify the 5–10% of symptomatic women with sinister underlying pathology [6]. The use of TVS as a triage tool exposes more than 50% of symptomatic women to further tests due to its low specificity [7]. Hysteroscopy and endometrial biopsy have high diagnostic accuracy but are invasive, anxiety provoking and painful; furthermore, the risk of technical failure is high [8]. Globally, millions of intrauterine investigations are carried out every year, with huge financial implications for health service providers [9] and at a significant personal cost to women [10]. An accurate, non-invasive, endometrial cancer detection tool that can triage symptomatic women for definitive testing while reassuring the large majority of women who do not have cancer would improve patient care [3].

The quest for simple, non-invasive, painless and convenient tests was voted the most important research priority for detecting cancer early by the James Lind Alliance, representing the views of patients, the general public and healthcare professionals [11]. Voided self-collected urine is an attractive biofluid for cancer biomarker discovery due to its simplicity, low-cost and ease of collection [12]. A urine test lends itself to self-sampling in community settings and is therefore likely to be acceptable to patients and clinicians alike [13]. The identification of endometrial cancer biomarkers in urine is dependent on renal excretion of systemic biomarkers or contamination of voided urine by uterine-derived biomarkers [12]. A urine test that accurately discriminates endometrial cancer from benign causes of PMB would represent a major advance in the field [14].

Recent progress in high-throughput and incisive technologies coupled with machine learning techniques have led to a new era of cancer biomarker discovery and validation [15]. Sequential window acquisition of all theoretical mass spectra (SWATH-MS), a data independent proteomic profiling platform, is a highly accurate and reproducible method for analysing biological samples and offers technological advantages for biomarker discovery due to its reproducibility, versatility, sensitivity and potential for data re-interrogation [16]. In this study, we carried out quantitative SWATH-MS-based proteomic profiling of urine acquired from a cohort of symptomatic women with and without endometrial cancer. Using machine learning techniques, robust predictive models for endometrial cancer detection were developed. This study provides proof of principle that a urine-based test could facilitate endometrial cancer detection and enable the effective triage of symptomatic post-menopausal women for urgent clinical diagnostics.

## Materials and methods

### Study hypothesis

Endometrial cancer biomarkers in urine may originate from two main sources: (i) the renal excretion of systemic cancer-related biomarkers and (ii) contamination of urine by naturally shed uterine-derived biomarkers due to the anatomical proximity of the urethra to the vagina [12]. We hypothesised that a two-pronged biomarker discovery approach, in which proteomic data files obtained from the SWATH-MS analysis of urine samples are searched against two spectral libraries (human plasma library and a bespoke endometrial cancer cervico-vaginal fluid library), has potential to deliver clinically relevant endometrial cancer biomarkers. This methodology is more likely to yield cancer-specific biomarkers than a urine-based spectral library, which would contain abundant nitrogenous waste products and urothelial proteins, leading to low cancer biomarker discovery rates. A urine-based spectral library is unlikely to contain endometrial cancer-derived proteins in the absence of direct tumour spread into the mucosal layer of the bladder, a rare and late event in endometrial cancer.

### Study participants

We recruited women with abnormal uterine bleeding, including those with known endometrial cancer attending the Gynaecology Outpatient Departments of St Mary’s Hospital, Manchester University NHS Foundation Trust and the Royal Oldham Hospital of the Northern Care Alliance NHS Group, between April 2019 and March 2020. Cases were confirmed to have endometrial cancer based on histological evaluation of biopsy or hysterectomy specimens, by at least two specialist gynaecological pathologists reporting to Royal College of Pathology Standards. Controls were women with no evidence of endometrial cancer or atypical hyperplasia, following routine diagnostic investigation for suspected endometrial cancer that included TVS, endometrial biopsy and/or hysteroscopy. Women with atrophic vaginitis, polyps and other benign conditions were eligible to serve as controls. We excluded women with a past history of gynaecological cancer and those without a uterus.

### Research sample and clinical data collection

Voided urine samples were self-collected in dry sterile urine collection pots. Samples were acquired prior to gynaecological examination and diagnostics (controls), or prior to treatment (cases). Samples were centrifuged at 1000 × *g* for 10 min at room temperature and the supernatant collected and stored at −80 °C, pending analysis. Demographic data, including age, body mass index, co-morbidities and medications used were recorded for all participants.

### Urine sample preparation

Urine supernatant samples were concentrated using the Agilent spin concentrator (4 mil 30 K MWCO concentrator, Agilent UK). Using the same spin, buffer exchange with 25 mM ammonium bicarbonate (ABC) was performed. Protein concentration was measured using the Bradford assay (Bio-rad laboratories, Watford, UK). Appropriate volumes of urine containing 50 µg of protein were transferred into clean Eppendorf vials. Disulfide bonds were reduced by the addition of 5 mM of dithiothreitol and 1% sodium deoxycholate to the fluid and incubation in a heating block at 60 degrees for 30 min. Alkylation was performed using 50 mM iodoacetamide in the dark at room temperature for 30 min and digestion completed with trypsin (Promega, Southampton, UK) at a 10:1 protein: trypsin ratio and incubated overnight at 37 °C. 1% Formic acid was then added to the sample for a final concentration of 0.5%. Deoxycholate was then pelleted by centrifugation at 12,000 × *g* for 10 min at 10 °C and the supernatant transferred to fresh microfuge tubes. Digested peptides were purified using SepPak C18 columns (Waters, Wilmslow, UK). Samples were then dried using the MiVac Quattro Concentrator for 3 h.

### SWATH-MS data acquisition

We carried out mass spectrometric analysis of the urine samples using a 6600 Triple TOF instrument (Sciex, Warrington, UK). The liquid chromatographic method was based on a 120-min gradient between a buffer A of 98% Water, 2% (v/v) Acetonitrile and 0.1% (w/v) Formic Acid and a buffer B of 80% Acetonitrile, 20% Water, 0.1% Formic Acid. Dried sample peptides were vigorously re-suspended in a buffer of 4% v/v Acetonitrile and 0.1% Formic Acid and injected in duplicate. We used an Eksigent system comprising of a nanoLC 400 autosampler along with a 425 pump module with YMC-Triart C18 trap column and a YMC-Triart C18 analytical column. We acquired mass spectra in SWATH mode and utilising the 100 variable window method with optimised collision energy equations. The spectral data files obtained were converted using wiffconverter (Sciex, Warrington, UK) and searched against an in-house plasma library (for systemic proteins with potential for excretion in urine) and our already published bespoke consensus spectral library for cervico-vaginal fluid proteins with potential to contaminate urine flow [17] using OpenSwath (version 2.0.0). We scored peptide matches using pyProphet (version 0.18.3) within the TransProteomic Pipeline (TPP) and subsequently aligned this using the TRIC tool from the OpenSWATH pipeline. The median coefficients of variation across technical replicates were <20 and were similar between cancers and controls. Researchers were blinded to clinical data and histological results during sample preparation and mass-spectrometric analyses. We performed downstream statistical analysis using the Bioconductor (release 3.5) packages SWATH2Stats and MSstats within the R language (version 3.4.1). We excluded all potential contaminants and decoy sequences prior to statistical analyses. The mass spectrometry proteomics data have been deposited to the ProteomeXchange Consortium via the PRIDE [18] partner repository with the data set identifier PXD038860.

### Data analysis

R version 4.1.1 (R Development Core Team, Vienna, Austria), STATA version 16, and MetaboAnalyst 4.0 were used for data analysis. Our power calculation confirmed that a sample size of 100 women, including *n* = 50 cases and *n* = 50 controls is needed to identify a (true) biomarker or biomarker panel that can detect endometrial cancer with an expected AUC of 0.90 (84, 96) at a 95% confidence level. We assessed data normality using the Shapiro–Wilk test and summarised continuous data using means (±standard deviation) or medians (IQR) as appropriate. Categorical data were summarised using counts (%). We assessed for differences between study groups using Student’s *t* test/Mann–Whitney *U* test for continuous variables and the chi-square test for categorical variables as indicated. Correction for multiple testing was performed using the Benjamini–Hochberg correction method (*q* = 0.05). The degree and direction of fold change between cases and controls was computed based on the ratio of protein concentrations, thus allowing for the identification of proteins with unidirectional alterations. We used principal component analysis (PCA) for dimensionality reduction and to visualise the degree of separation between study groups. Hierarchical clustering (heat maps) was performed using the Euclidean distance measure and the Ward algorithm. Random Forest (RF) machine learning technique was used to identify the most important discriminatory classifiers which were subsequently ranked according to the mean decrease accuracy metric and the mean decrease in Gini index. The discriminatory biomarkers were used to create nested logistic regression models based on parsimony and predictive accuracy. We used the forward stepwise regression approach to develop models of increasing complexity by the sequential addition of discriminatory proteins. The order of protein inclusion was based on their ranking in the RF accuracy metric. We made an a priori decision to limit the maximum number of proteins included in the final model to 10, thus ensuring simplicity of the parsimonious model. The performance of this model was assessed by the area under the receiver characteristic curve (AUC) and the 95% confidence intervals (CIs). Likelihood ratio tests were used to compare nested model performance. Interactions were tested within the regression framework by the introduction of first-order interaction terms.

## Results

### Participant demographics

In total, 104 women participated in this study, including 50 (48.1%) with a histologically confirmed diagnosis of endometrial cancer and 54 (51.9%) with no evidence of cancer (Table 1). Their median age and BMI were 57 years (interquartile range (IQR) 52, 68) and 29 kg/m^2^ (IQR 24, 34), respectively, and they were mostly of White British ethnicity (86.5% White, 10.7% Asian and 2.9% Afro-Caribbean). Women with endometrial cancer were older (median age 65 years (IQR 57, 73) vs 53 years (IQR 51, 58), *p* < 0.001) and with higher BMI (median 30 kg/m^2^ (IQR 26, 37) vs 27 kg/m^2^ (IQR 23, 33), *p* = 0.020), than their control counterparts (Table 1). Most of the cases had low-grade (64% grade I/II), early-stage (86% FIGO stage I/II) endometrial cancer of endometrioid histological subtype (82%). Twenty tumours (40.0% of cases) showed lymphovascular space invasion and 21 (42.0%) ≥50% myometrial invasion. The controls were mostly symptomatic from vulvovaginal atrophy (20.4%), benign endometrial or endocervical polyps (30.6%). However, in almost 50% of cases, no underlying pathology was found.

**Table 1:** Clinico-pathological characteristics of the study cohort.

Demographic characteristics of study population				
	Total cohort (104)	Cases (50, 48.1%)	Controls (54, 51.9%)	*p* value
*Patient characteristics*				
Age (years) median (IQR)	57 (52, 68)	65 (57, 73)	53 (51, 58)	<0.001
BMI (kg/m^2^) median (IQR)	29 (24, 34)	30 (26, 37)	27 (23, 33)	0.020
White ethnicity	90 (86.5%)	43 (86.0%)	47 (87.0%)	0.877
History of diabetes mellitus	15 (14.4%)	12 (24.0%)	3 (5.6%)	0.002
*Tumour characteristics*				
FIGO grade (2009)				
Grade 1/2	–	32 (64.0%)	–	
Grade 3	–	18 (36.0%)	–	
FIGO stage (2009)				
Stage 1/2	–	43 (86%)	–	
Stage 3/4	–	7 (14%)	–	
Histological phenotype				
Endometrioid	–	41 (82.0%)	–	
Non-endometrioid	–	9 (18.0%)	–	
Myometrial invasion ≥50%	–	21 (42.0%)	–	
Presence of LVSI	–	20 (40.0%)	–	

### Renally excreted systemic biomarkers

**Fig. 1:**
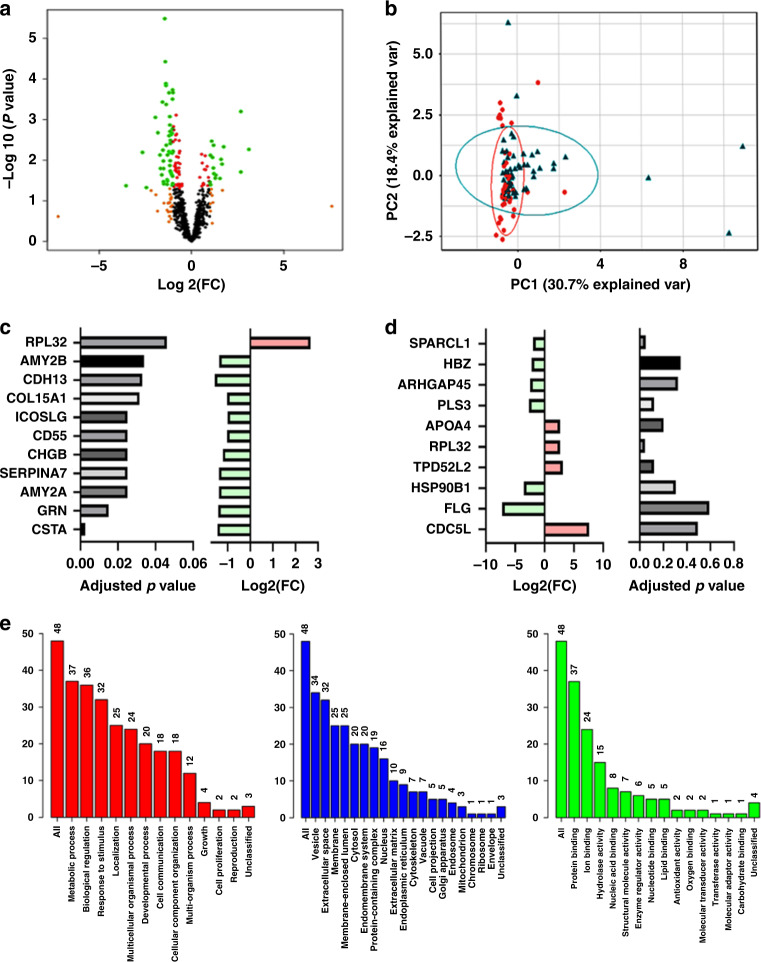
Exploratory differential and gene ontological analyses of renally excreted urinary protein biomarkers for endometrial cancer detection. **a** Volcano plot showing protein distribution by log fold change and degree of significance, **b** PCA showing pattern of separation based on the top 10 discriminatory proteins identified by Random Forest machine learning technique, **c** histogram showing discriminatory proteins with a statistically significant adjusted *p* (Benjamini correction) and their the associated degree of fold change, **d** histogram showing the discriminatory proteins with the largest range of fold change and their associated adjusted *p* values, **e** gene ontology analyses of the discriminatory proteins showing a log2 fold change of at least 1.2 mapped using the WebGestalt Webserver. Biological processes (Red), cellular processes (Blue) and molecular functions (Green) are shown. The height of each bar represents the number of mapped proteins per category.

**Fig. 2:**
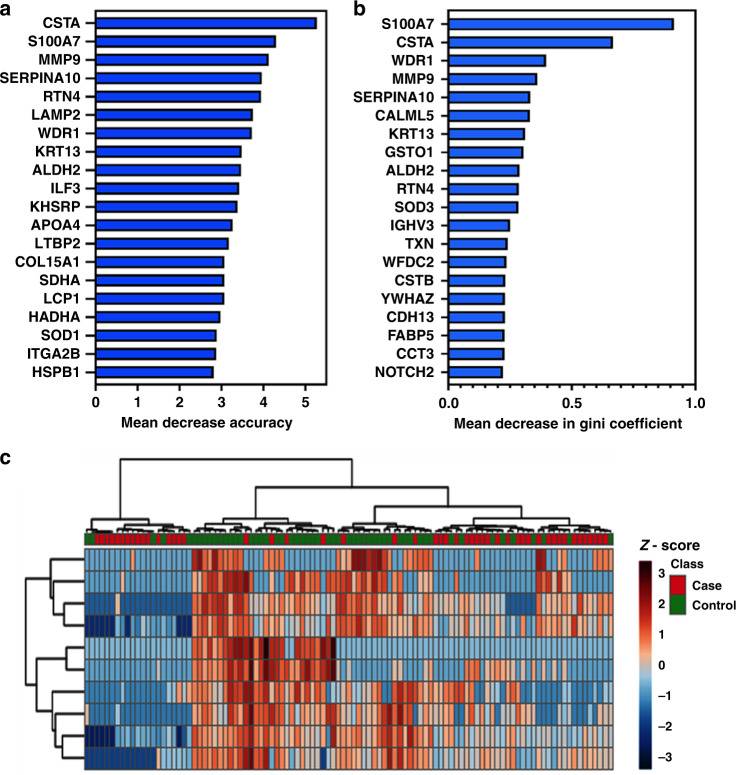
Ranking and hierarchical clustering using the top discriminatory renally excreted urinary protein biomarkers for endometrial cancer detection. **a**, **b** Top 20 discriminatory renally excreted proteins identified by Random Forest machine learning technique and ranked by their contribution to classification accuracy using the mean decrease accuracy (**a**) and mean decrease Gini index (**b**) based on all study samples. **c** Hierarchical clustering using the top discriminatory proteins. The difference in intensities of the biomarkers by cancer-control status is shown. Each coloured cell in the map represents scaled/relative concentration of the indicated protein. Proteins are clustered along the vertical axis while subjects are clustered along the horizontal axis. Hierarchical clustering was based on the Euclidean distance measure and Ward algorithm

Self-collected voided urine samples were processed as described and the resulting data searched against the human plasma library to identify renally excreted endometrial cancer-related systemic biomarkers. A total of 798 urinary proteins were quantified across all samples. An exploratory analysis based on differential expression is summarised in Fig. 1a. PCAs using the top 10 discriminatory proteins by the RF model showed minimal separation between the cancers and controls (Fig. 1b). A total of 49 proteins were observed to have a log2 fold change >1.2 when comparing cancers to controls. Of these, 39 were statistically significant (*p* < 0.05) and 11 proteins were significant following multiple testing corrections (adjusted *p* < 0.05) (Fig. 1c). The most significant of the differential proteins was Cystatin A (CSTA) (Fig. 1c). The proteins exhibiting the greatest log2 fold change and their adjusted *p* values are summarised in Fig. 1e. Cell division cycle 5-like protein (CDC5L) and Filaggrin (FLG) exhibited the largest fold changes, being sevenfold higher and lower in endometrial cancer compared to controls, respectively. Tumour protein D54 (TPD52L2) had a threefold higher concentration in endometrial cancer compared to controls while the molecular chaperone endoplasmin (HSP90B1) had a threefold lower concentration (Fig. 1d). Gene ontology analyses of the differential proteins (Log FC > 1.2) confirmed these proteins to have metabolic and biological regulatory functions and to originate from the extracellular space/vesicles and with protein and ion binding activities (Fig. 1e). The top 20 discriminatory proteins identified by RF machine learning were subsequently ranked by their contribution to the classification accuracy based on the mean decrease accuracy metric (Fig. 2a) and the mean decrease Gini index (Fig. 2b). Hierarchical clustering confirmed the moderate ability of identified proteins to discriminate between cancers and controls (Fig. 2c).

Classical univariate ROC curve analyses of important discriminatory biomarkers was subsequently carried out to assess their ability to separate endometrial cancer from controls. In this analysis, Calcium Binding Protein A7 (S100A7) and Cystatin-A (CSTA) were found to predict cancer with AUC values of 0.77 (95% CI 0.67–0.85) and 0.77 (95% CI 0.66–0.86), respectively (Fig. S1). Other discriminatory biomarkers included Fatty acid-binding protein 5 (FABP5), Thioredoxin (TXN) and Cell adhesion molecule 1 precursor (CADM1) with AUC values of 0.73 (95% CI 0.64–0.82), 0.73 (0.63–0.83) and 0.72 (95% CI 0.62–0.80), respectively. An adjusted (Turkey) box-plot analyses of important proteins is presented in Fig. S2.

**Fig. 3:**
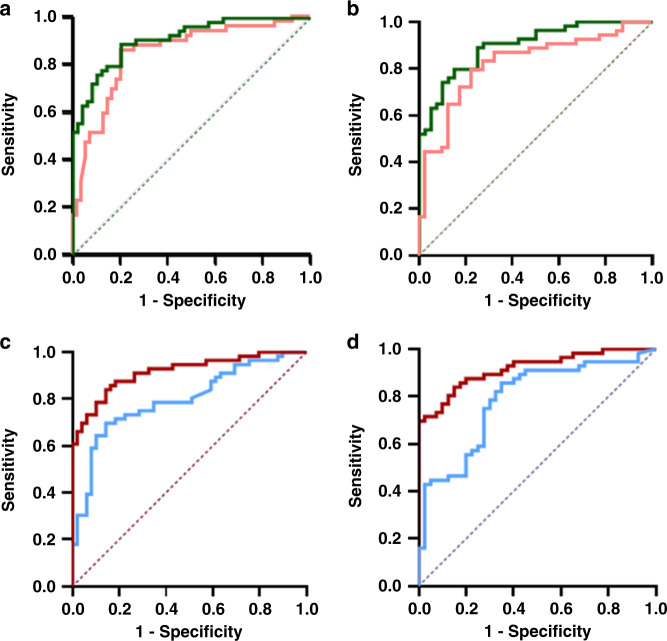
Multimarker receiver operating characteristic (ROC) curve analyses. ROC curves for combined biomarker panels based on renally excreted biomarkers for the detection of all cancers (**a**) and early-stage cancers (**b**). Green represents 10-marker panel and Pink 4-marker panel. ROC curves for combined biomarker panels based on uterine-derived biomarkers for the detection of all cancers (**c**) and early-stage cancers (**d**). Red represents a ten-marker panel and Blue a four-marker panel.

Next, we used forward stepwise logistic regression modelling to create nested models of increasing complexity. The sequential addition of discriminatory proteins improved predictive accuracy. A four-marker panel comprising CSTA, S100A7, MMP9 and SERPINA10 predicted endometrial cancer with an AUC of 0.84 (0.77–0.92), sensitivity of 72.2%, specificity of 87.8%, NPV of 86.7% and PPV of 74.1% (Fig. 3a). The incorporation of RTN4, LAMP2, WDR1, KRT13, ALDH2 and ILF3 gave rise to a ten-marker biomarker panel, which predicted endometrial cancer with an AUC of 0.91 (0.86–0.96), and was the best performing diagnostic model (Fig. 3a). This model was adjudicated to be the parsimonious model and demonstrated a sensitivity, specificity, NPV and PPV of 79.6, 83.7, 84.3 and 78.9%, respectively (Table S1).

It is crucial that urine protein biomarkers for use in clinical practice are able to detect early-stage (FIGO stage I/II) endometrial cancer and not just advanced stage disease. We therefore sought to identify urine proteins that can discriminate early-stage (FIGO stage I/II) endometrial cancer from controls. Based on this analysis, CSTA and S100A7 were confirmed to discriminate early-stage endometrial cancer from controls with AUC values of 0.75 (0.64–0.85) and 0.73 (0.63–0.83), respectively. Other important discriminatory proteins were Proteasome subunit beta type-7 (PSMB7) and TXN, with AUC values of 0.73 (0.63–0.83) and 0.72 (0.61–0.82), respectively. The four-biomarker panel comprising S100A7, CSTA, MMP9 and SERPINA 10 predicted early-stage cancer with AUC 0.83 (0.74–0.92) (Fig. 3b) while the equivalent ten-marker panel additionally incorporating RTN4, LAMP2, WDR1, KRT13, ALDH2 and ILF3 predicted early-stage endometrial cancer with AUC of 0.90 (0.84–0.96) (Fig. 3b and Table S2).

### Uterine-derived protein biomarkers

**Fig. 4:**
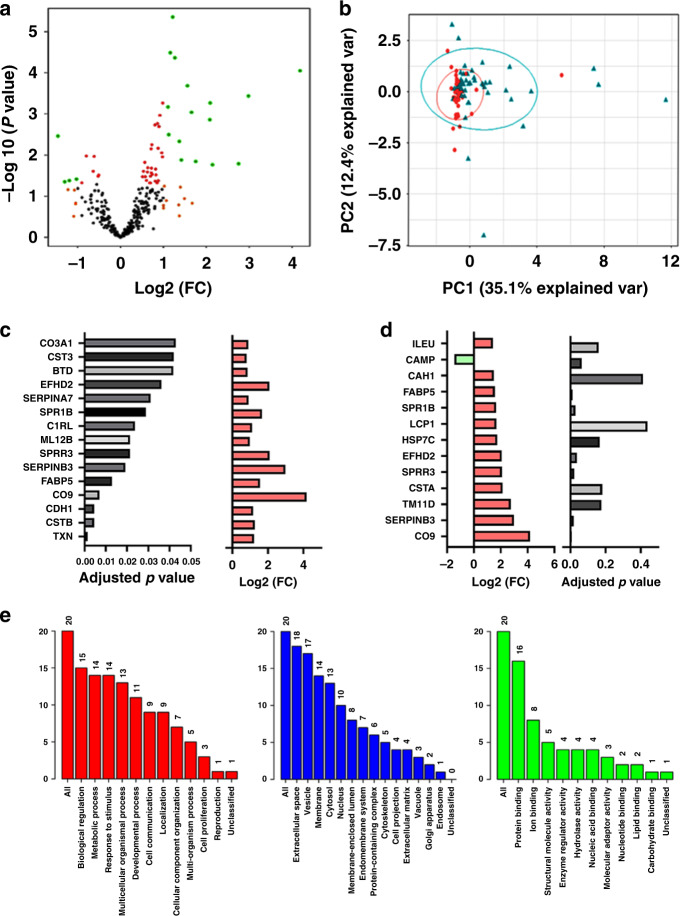
Exploratory differential and gene ontological analyses of uterine-derived protein biomarkers contaminating urine. **a** Volcano plot showing protein distribution by log fold change and degree of significance. **b** PCA showing pattern of sample separation based on the top 10 discriminatory proteins identified by Random Forest machine learning technique. **c** Histogram showing discriminatory proteins with a statistically significant adjusted *p* value by Benjamini correction and the associated degree of fold change. **d** Histogram showing the discriminatory proteins with the largest range of fold change and their associated adjusted *p* values. **e** Gene ontological analyses of the discriminatory proteins showing a log2 fold change of at least 1.2 mapped using the WebGestalt Webserver. The biological processes (Red), cellular processes (Blue) and molecular functions (Green) are shown. The height of each bar represents the number of mapped proteins per category.

**Fig. 5:**
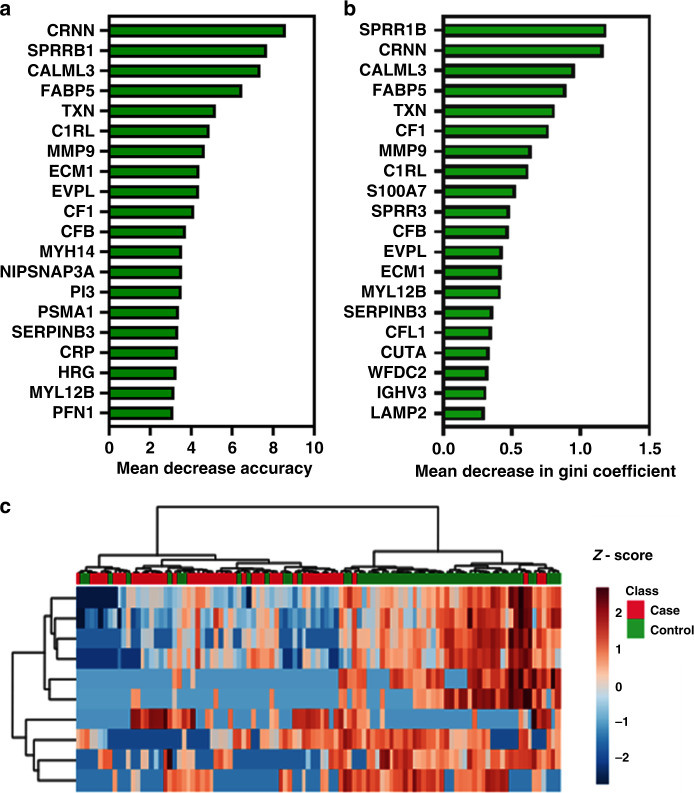
Ranking and hierarchical clustering using the top discriminatory uterine-derived proteins contaminating urine. **a**, **b** Top 20 discriminatory uterine-derived proteins identified by Random Forest machine learning technique and ranked by their contribution to classification accuracy using the mean decrease accuracy (**a**) and mean decrease Gini index (**b**) based on all study samples. **c** Hierarchical clustering using the top discriminatory proteins. The difference in intensities of the biomarkers by cancer–control status is shown. Each coloured cell in the map represents scaled/relative concentration of indicated protein. Proteins are clustered along the vertical axis while subjects are clustered along the horizontal axis. Hierarchical clustering was based on the Euclidean distance measure and Ward algorithm.

To identify uterine-derived biomarkers contaminating urine, we searched the obtained urine proteomic data sets against our previously published endometrial cancer cervico-vaginal fluid spectral library [17]. A total of 316 proteins were quantified across all study samples using the bespoke cervico-vaginal fluid (CVF) library. An exploratory analysis based on differential expression is summarised in Fig. 4a. Principal components analyses using the top 10 discriminatory proteins by the RF model is presented in Fig. 4b. A total of 21 proteins were observed to have a log2 fold change greater than 1.2 when comparing cancers to controls. Of these, 18 were statistically significant (unadjusted *p* < 0.05) and eight remained significant following multiple testing corrections (adjusted *p* < 0.05). The most significant of the differentially expressed proteins following multiple testing corrections was Thioredoxin (TXN) (Fig. 4c). The proteins exhibiting the greatest log2 fold change and their adjusted *p* values are summarised in Fig. 4d. Complement component C9 (CO9) exhibited the largest fold change, being fourfold higher in endometrial cancer compared to controls. SerpinB3 and transmembrane protease serine 11D (TM11D) were almost threefold higher in endometrial cancer respectively (Fig. 4d). Gene ontological analyses of the 21 differential proteins (Log FC > 1.2) confirmed these proteins to have biological regulatory and metabolic functions and to originate from the extracellular space and with protein and ion binding activities (Fig. 4e). The top 20 discriminatory proteins identified by RF machine learning were subsequently ranked by their contribution to the classification accuracy based on the mean decrease accuracy metric (Fig. 5a) and the mean decrease Gini index (Fig. 5b). Hierarchical clustering confirmed the moderate ability of identified proteins to discriminate between cancers and controls (Fig. 5c).

Classical univariate ROC curve analyses some of the important discriminatory biomarkers selected by the mean decrease accuracy metric of the RF model is as presented in Fig. S3 and Fig. 5. SPRR1B was the best performing biomarker with an AUC of 0.79 (0.69–0.88). CRNN, CALML3 and TXN predicted endometrial cancer with AUC values of 0.75 (0.64–0.84), 0.75 (0.65–0.84) and 0.75 (0.66–0.85) respectively. An adjusted (Tukey) box-plot analyses showed evidence of lower concentrations of SPRR1B, CRNN, TXN and FABP5 in cancers compared to controls (Fig. S4).

Next, we used multiple logistic regression analysis in a forward stepwise approach to assess the performance of combinations of the top discriminatory biomarkers. A four-marker panel comprising of SPRR1B, CRNN, CALML3 and TXN predicted endometrial cancer with an AUC of 0.80 (0.71–0.88) (Fig. 3c). The sensitivity and specificity of this panel was 89.8% and 64.3%, respectively, while the PPV and NPV was 88.0% and 69.0%, respectively. The best performing multi-marker signature combined the four biomarker panel with FABP5, C1RL, MMP9, ECM1, S100A7 and CF1. This signature predicted endometrial cancer with an AUC of 0.92 (0.86–0.97), sensitivity of 83.7% and specificity of 83.9% and was adjudicated to be the parsimonious model (Fig. 3c and Table S1). The PPV and NPV for this panel were 85.5% and 82.0%, respectively.

Next, we sought to identify uterine-derived urinary biomarkers for the detection of early-stage endometrial cancer. In this analysis, SPR1B and TXN were important discriminators with AUC values of 0.78 (0.68–0.87) and 0.77 (0.67–0.86), respectively. Other important biomarkers for the detection of early-stage disease were CRNN and CALM-3, with AUC values of 0.75 (0.66–0.84) and 0.75 (0.67–0.83), respectively. The four-marker panel comprising SPR1B, TXN, CRNN and CALM3 predicted endometrial cancer with an AUC of 0.78 (0.69–0.88). The 10-biomarker signature in this category additionally incorporating FABP5, C1RL, MMP9, ECM1, S100A7 and CF1, discriminated early-stage cancers from controls with an AUC of 0.92 (0.86–0.97) (Fig. 3d and Table S2).

## Discussion

### Main findings

In this study, we explored the potential of urine-based protein biomarkers to detect endometrial cancer in a cohort of symptomatic women. Using a novel approach, we identified systemic cancer biomarkers that are excreted by the kidneys as well as those potentially originating from the tumour and shed down the lower genital tract, contaminating urine. The top discriminatory biomarkers individually showed moderate accuracy (AUC > 0.70) for endometrial cancer detection overall. However, algorithms combining the most discriminatory proteins were more successful, with AUCs > 0.90. The best performing diagnostic model was a 10-marker panel combining SPRR1B, CRNN, CALML3, TXN, FABP5, C1RL, MMP9, ECM1, S100A7 and CF1 and predicted endometrial cancer with an AUC of 0.92, sensitivity of 83.7% and specificity of 83.9%. These data suggest that a urine-based test could offer a minimally invasive endometrial cancer triage tool in symptomatic women and confirmation in a larger independent cohort is warranted.

### Strengths and limitations

To our knowledge, this is the first study exploring the use of SWATH-MS-based proteomics for the identification of endometrial cancer biomarkers in urine. The use of SWATH-MS is a major strength of our study, as it is a proteomic platform with high precision, accuracy and reproducibility [16]. The exploitation of urine, the prototype non-invasive sample, is another strength of our study as self-collection of voided urine is highly acceptable to women, offering opportunities for community-based collection and repeat sampling [12]. The biomarkers identified in this study showed good accuracy to warrant clinical translation, especially when combined in a multi-marker panel. Indeed, several of the identified biomarkers have mechanistic links with the malignant transformation process. The choice of our control group, i.e. symptomatic women who do not have endometrial cancer, is yet another strength, as these are the women for whom the new detection tool is intended. All cases were confirmed to have endometrial cancer based on gold-standard histopathological evaluation, while the controls were excluded as cancer cases using standard clinical diagnostic pathways and followed up for 12 months, thus alleviating concerns about misclassification bias. Our approach of searching the urine proteomic data sets against the plasma and cervico-vaginal fluid spectral libraries allowed for the identification of renally excreted systemic biomarkers and uterine biomarkers shed from the lower genital tract into urine. This approach is more likely to yield clinically relevant biomarkers compared with a search of a urine-based library, which would largely be dominated by nitrogenous waste products and urothelial proteins. We made an a priori decision to limit the maximum number of classifiers in the diagnostic models to 10, thus ensuring future validated assay simplicity. While a more complex modelling approach with the inclusion of >10 classifiers may lead to better diagnostic accuracy, it is unlikely to be easily translated to routine clinical practice. Limitations of our study include the relatively small numbers of the various endometrial cancer subtypes and stages, which precluded an assessment of the performance of the biomarker signatures for the detection of advanced stage and biologically aggressive endometrial cancer phenotypes. We do not know how well the identified biomarkers will perform in pre-menopausal women, or whether they could be used as a screening tool in asymptomatic women of the general population or in women with a hereditary predisposition to endometrial cancer (e.g. Lynch syndrome). Furthermore, the exploitation of urine for biomarker discovery may be confounded by hydration status, medications used, renal function and diet [12]. As such, further work is needed in terms of standardising sample collection and processing, before urine-based assays can be introduced for endometrial cancer detection in routine clinical practice. Finally, the use of SWATH-MS as an analytic tool for endometrial cancer detection is presently impractical for use in clinical settings. It is feasible that Selected Reaction Monitoring mass spectrometry may become sufficiently automated and sensitive in the future [19], however there is a need for robust, clinically tractable urine biomarker assays using platforms like ELISA or lateral flow test technology with validated reproducibility for translation.

### Interpretation

Only a few studies have explored the potential of urine for endometrial cancer biomarker discovery. Proteins, metabolites, micro-RNA and cytology have all been explored as endometrial cancer biomarker targets [12, 14, 20–23]. As yet, there is limited evidence to enable clinical translation. Most of the previous studies have been small pilot studies based on analytical platforms that lack precision and reproducibility [12]. Mu and colleagues used two-dimensional gel electrophoresis and LC-MS/MS to characterise urine samples acquired from women with early stage endometrial cancer (*n* = 7) and age-matched controls (*n* = 11) and found altered levels of zinc alpha-2-glycoprotein, alpha-1-acid glycoprotein and CD59 in endometrial cancer cases compared to controls [24] in a study limited by analytical sensitivity and protein quantification. In another small study by the same group, a glycopeptide with mass/charge ratio of 1449 of unknown origin was found to discriminate endometrial cancer from controls based on proteomic analysis of urine using surface enhanced laser desorption/ionisation time-of-flight (SELDI-TOF) [25]. Bazzett and colleagues found no evidence of an association between endometrial cancer and urinary MMP [26] while Mattila et al. reported an upregulation of epidermal growth factor (EGF) in the urine of women with endometrial cancer [27]. A more recent study by Stockley et al. found urine sediment MCM5 discriminated endometrial cancer from benign disease with an AUC of 0.83 [28]. This single biomarker test is now being validated in a large multicentre study across several international sites.

To our knowledge, our study is the largest to investigate urine proteins for endometrial cancer detection. We show evidence that urine protein biomarkers can identify endometrial cancer with sufficient accuracy to warrant clinical translation, especially when combined in a multi-marker panel. The top discriminatory biomarkers demonstrating mechanistic links with endometrial tumourigenesis were CRNN and CSTA. Individually, CRNN and CSTA predicted endometrial cancer with AUC values of 0.75 and 0.77 respectively. CRNN is pro-proliferative and plays an important role in the GI/S cell cycle progression by inducing expression of the cell cycle regulator CCND1 [29]. Importantly, CRNN regulates malignant cell proliferation induced by pro-inflammatory cytokine response by activating NFB1 and P13K/AKT signalling pathways, both of which are implicated in endometrial carcinogenesis [29]. Cystatins, on the other hand, are cysteine protease inhibitors that play crucial roles in cell proliferation, differentiation, invasion, angiogenesis and immunomodulation [30]. Mechanistic studies have been consistent in suggesting the potential role of cystatins in cancer development and progression [30]. Our finding of lower levels of cystatin A in the urine of women with endometrial cancer is consistent with previous work where cystatin A has been reported to act as a tumour suppressor in oesophageal and lung cancers [31]. The study by Ma and colleagues concluded that cystatin A exerts a tumour suppressive effect by inhibiting MAPK and AKT pathways [31]. In addition, cystatin A gene silencing is associated with epigenetic regulation while its overexpression facilitates mesenchymal-to-epithelial transition [31].

Other discriminatory proteins with moderate accuracy (AUC > 0.70) include FABP5, MMP9 and SERPINA10. These biomarkers have previously been reported to have potential as endometrial cancer biomarkers in tissue and plasma samples [32]. FABP5 plays crucial roles in cell signalling and modulates gene expression and cell differentiation [33]. MMPs degrade extracellular matrix proteins during tissue growth and turnover. Soini and colleagues, in an analysis of the messenger RNA of MMP-9, concluded that MMP-9 was differentially expressed in hyperplastic endometrium compared to non-neoplastic endometrium [34], thus suggesting a potential role in endometrial carcinogenesis. SERPINA 10 (protein Z) is a serine proteinase inhibitor previously reported to be reduced in endometrial cancer [35]. A previous study from our group found evidence for its potential utility as a serum biomarker for ovarian cancer detection [36]. SERPINA 10 is predominantly expressed in the liver and secreted in plasma. Therefore, its identification in urine is most likely a result of its excretion by the kidneys. These biomarkers need to be externally validated and their roles in endometrial carcinogenesis elucidated prior to clinical implementation.

Urine SPRR1B, S100A7, CALML3 and TXN were also found to have potential as endometrial cancer biomarkers. SPRR1B is a proline-rich molecule that has been associated with cervical intraepithelial neoplasia [37]. S100A7 plays a crucial role in the innate immune system and has been linked to bladder squamous cell carcinoma [38]. CALML3 has calcium binding and enzyme regulatory functions and plays a role in B cell receptor signalling pathways [39] while TXN plays crucial roles in redox reactions [40]. The combined biomarker panels in this study showed improved ability to discriminate endometrial cancer from controls when compared to single biomarker candidates.

### Conclusion

In conclusion, a urine-based assay could offer a patient-friendly endometrial cancer detection tool that can be used in the community to triage symptomatic women for definitive diagnostic testing. Our multi-marker panels demonstrated sufficient accuracy (AUC > 0.90) to warrant clinical utility and several of the identified biomarkers have mechanistic links with the malignant transformation process. Large multi-centre prospective studies are now needed to confirm these findings and to fully elucidate the role of the identified biomarkers in endometrial tumourigenesis.

## Supplementary information

Supplementary Data

Tables S1 and S2

Figure S1

Figure S2

Figure S3

Figure S4

## Data Availability

The mass spectrometry proteomics data have been deposited to the ProteomeXchange Consortium via the PRIDE partner repository with the data set identifier PXD038860.

## Code availability

Code script files can be provided upon reasonable request.
