# Supplementary material for: Quantitative SWATH-based proteomic profiling of urine for the identification of endometrial cancer biomarkers in symptomatic women
Source: Br J Cancer. 2023 Feb 17;128(9):1723–32. doi: 10.1038/s41416-022-02139-0 (PMC10133303; doi:10.1038/s41416-022-02139-0)
Supplement: Supplementary file 1 — Supplementary Data [file 41416_2022_2139_MOESM1_ESM.docx]

**Supplementary data**

**Figure S1:** ROC curve analyses of promising endometrial cancer diagnostic biomarkers AUC: Area under the Curve.

**Figure S2:** Adjusted (Tukey) box plot distribution of selected discriminatory renally excreted biomarkers. The notches represent the 95% confidence interval around the median concentration of each marker for the group.

**Figure S3:** Receiver operating characteristic (ROC) curve analyses of the promising endometrial cancer diagnostic biomarkers. AUC: Area under the Curve.

**Figure S4:** Adjusted (Tukey) box plot distribution of selected discriminatory uterine-derived biomarkers. The notches represent the 95% confidence interval around the median concentration of each marker for the group.

**FigS1**

**
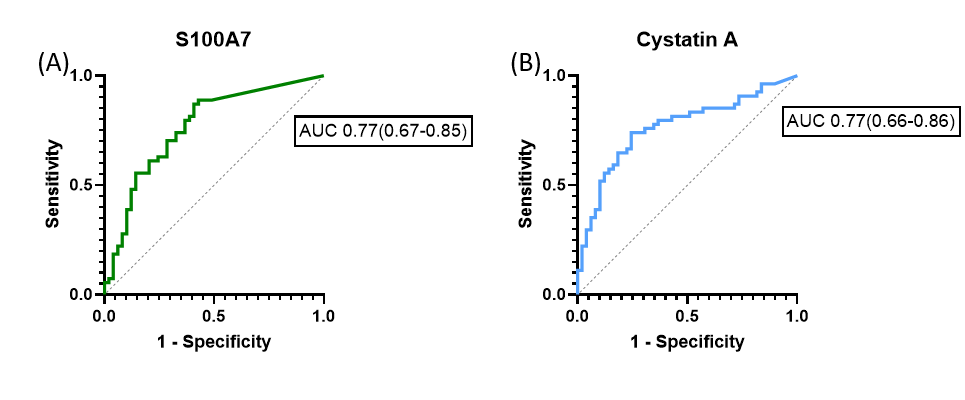
**

**FigS2**

**
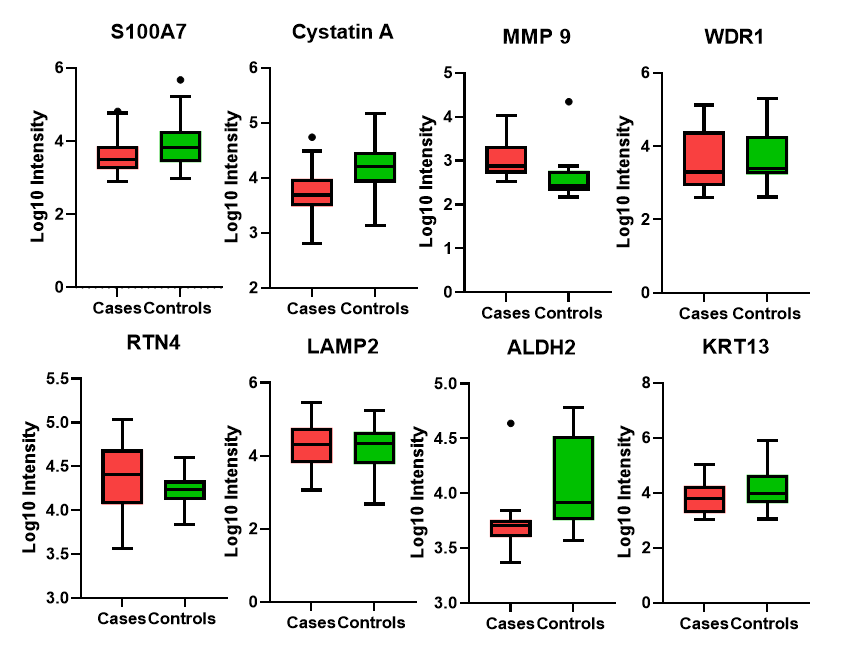
**

**Fig S3**

**
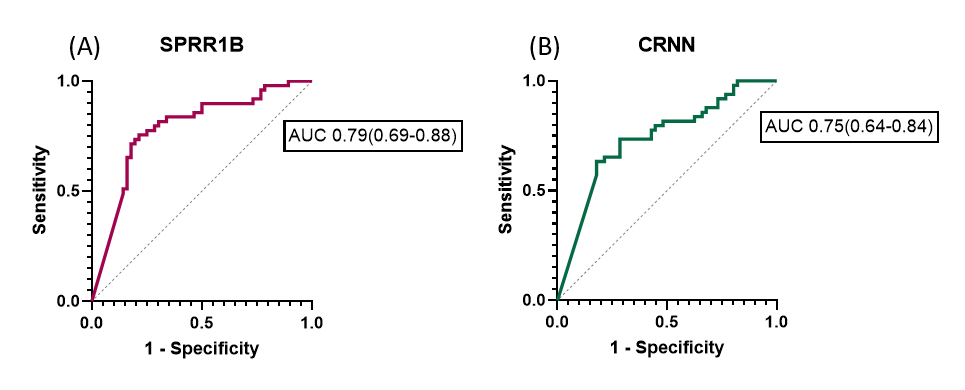
**

**Fig S4**

**
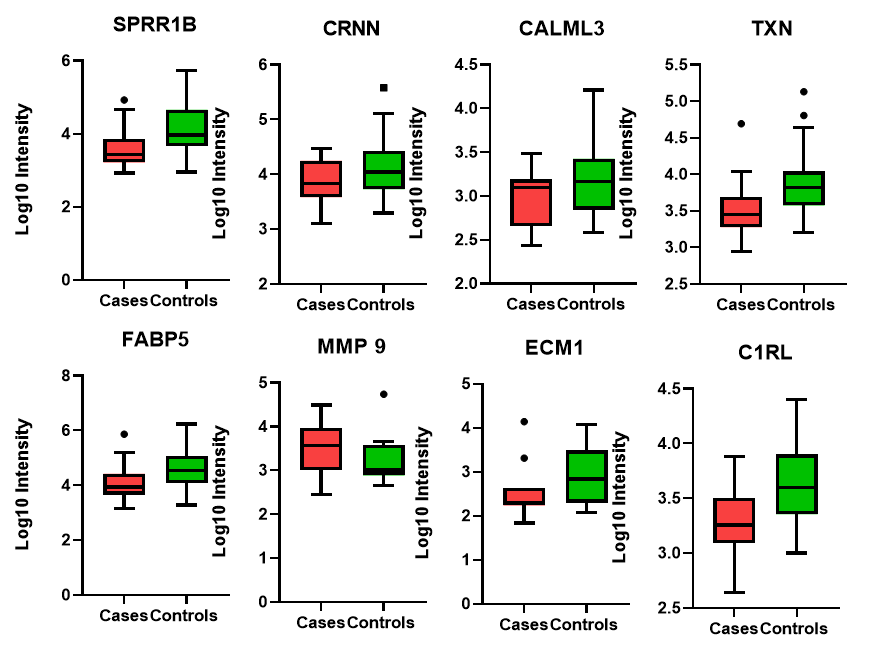
**

**TABLE S1**: Parsimonious urine biomarker models for endometrial cancer detection

| **Renally excreted protein biomarkers** | | | | | |
| --- | --- | --- | --- | --- | --- |
| **Panel** | **AUC(95%CI)** | **SEN** | **SPE** | **PPV** | **NPV** |
| **S100A7**+CSTA+**MMP9**+SERPINA10+RTN4+LAMP2+WDR1+  KRT13+ALDH2+ILF3 | 0.91 (0.86-0.96) | 79.6% | 83.7% | 78.9% | 84.3% |
| **Uterine derived biomarkers** | | | | | |
| **Panel** | **AUC(95%CI)** | **SEN** | **SPE** | **PPV** | **NPV** |
| SPRR1B+CRNN+CALML3+TXN+FABP5+C1RL+**MMP9** +ECM1+**S100A7** + CF1 | 0.92(0.86-0.97) | 83.7% | 83.9% | 85.5% | 82.0% |

**Table S2:** Parsimonious urine biomarker models for the detection of early stage endometrial cancer

| **Renally excreted protein biomarkers** | | | | | |
| --- | --- | --- | --- | --- | --- |
| **Panel** | **AUC(95%CI)** | **SEN** | **SPE** | **PPV** | **NPV** |
| S100A7+CSTA+MMP9+SERPINA10+RTN4+LAMP2+WDR1+  KRT13+ALDH2+ILF3 | 0.90 (0.84-0.96) | 87.0% | 75.0% | 82.5% | 81.1% |
| **Uterine derived biomarkers** | | | | | |
| Panel | **AUC(95%CI)** | **SEN** | **SPE** | **PPV** | **NPV** |
| SPRR1B+CRNN+CALML3+TXN+FABP5+C1RL+MMP9 +ECM1+S100A7 + CF1 | 0.92(0.86-0.97) | 80.0% | 87.5% | 82.1% | 86.0% |
