# Supplementary figures and images for: Quantitative SWATH-based proteomic profiling of urine for the identification of endometrial cancer biomarkers in symptomatic women
Source: Br J Cancer. 2023 Feb 17;128(9):1723–32. doi: 10.1038/s41416-022-02139-0 (PMC10133303; doi:10.1038/s41416-022-02139-0)

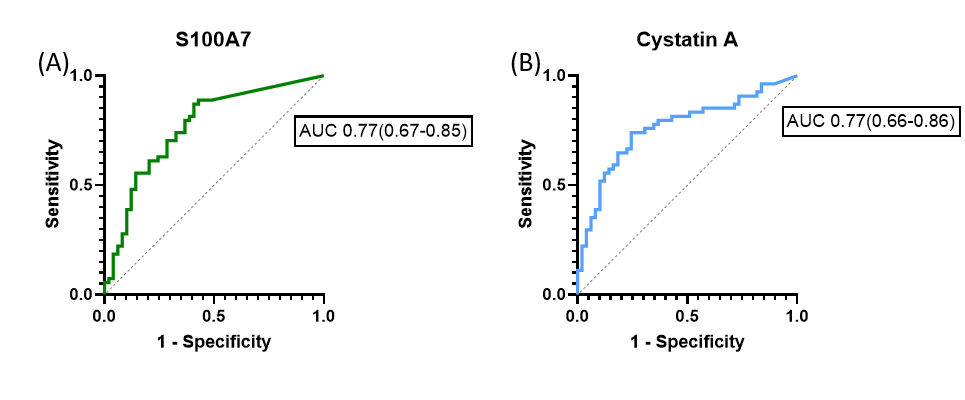

Supplement: Supplementary file 3 — Figure S1 [file 41416_2022_2139_MOESM3_ESM.png]

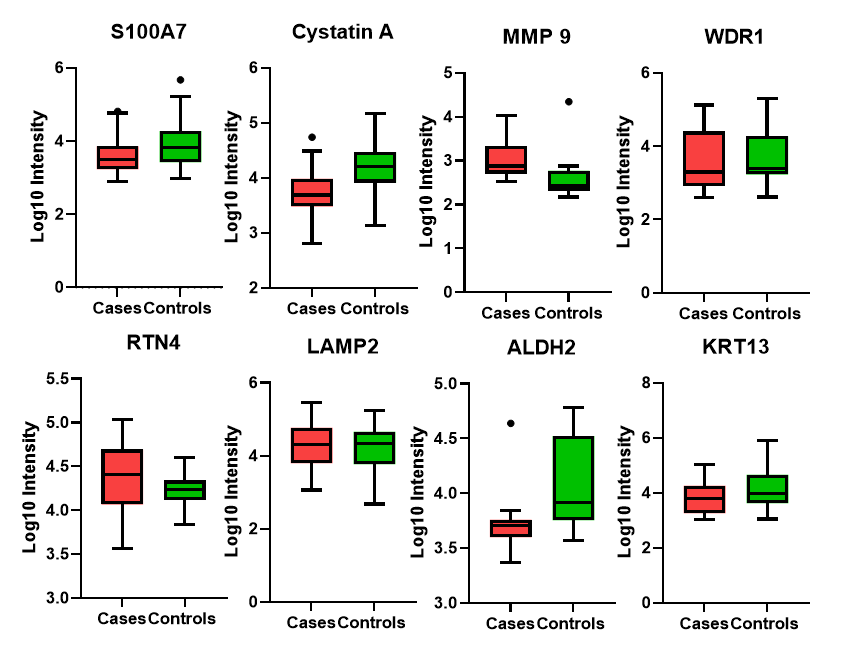

Supplement: Supplementary file 4 — Figure S2 [file 41416_2022_2139_MOESM4_ESM.png]

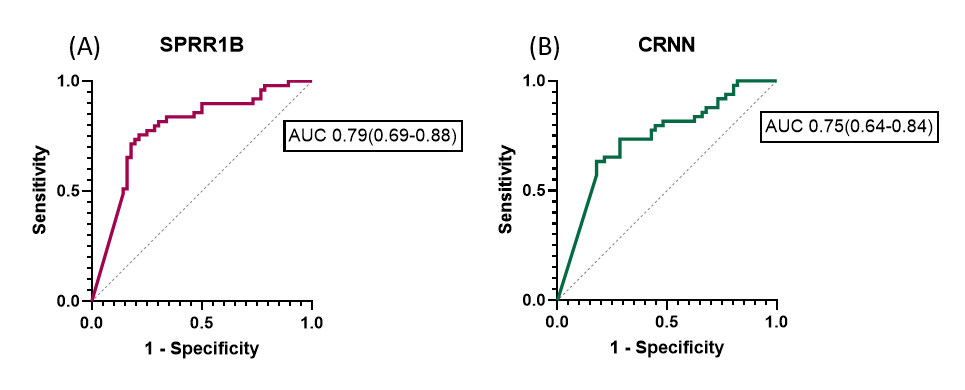

Supplement: Supplementary file 5 — Figure S3 [file 41416_2022_2139_MOESM5_ESM.png]

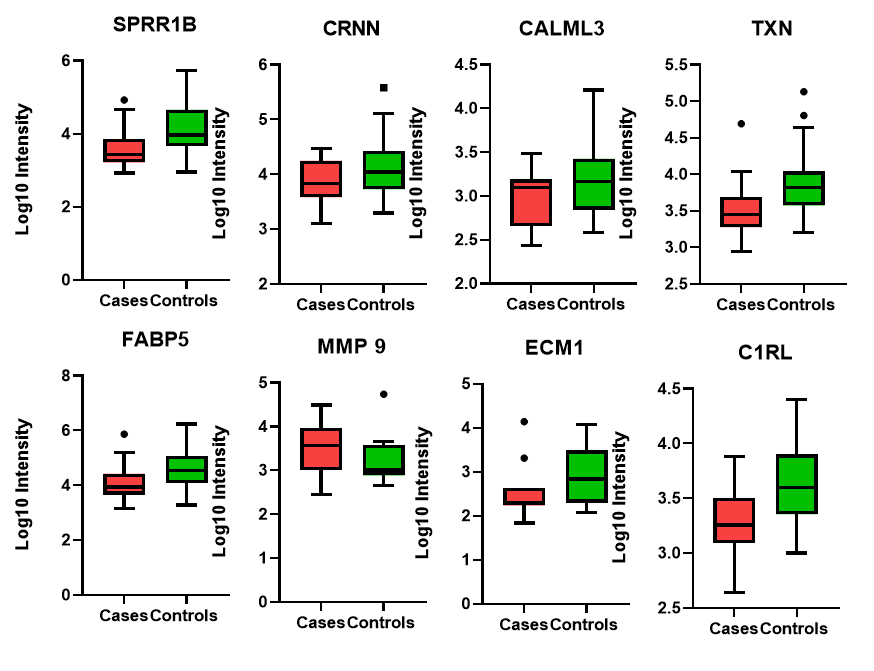

Supplement: Supplementary file 6 — Figure S4 [file 41416_2022_2139_MOESM6_ESM.png]
